# Dr. Dowler

**Published:** 1849-02

**Authors:** 


*Dr. Dowler.*—We observe an announcement in some of our exchanges, of the election of Bennet Dowler, M. D., of New Orleans, to honorary membership of the Philadelphia Academy of Arts and Sciences. This is a merited honor. Dr. D. has contributed scientific observations and experiments, on various subjects, alike original, curious and important. We have not met with any communications from his pen of late. But his mind is too actively bent on investigations calculated to extend our knowledge over the domains of natural phenomena, to rest satisfied with what he has already achieved. The medical public will hear from him anon.

